# Sex, Receptors, and Attachment: A Review of Individual Factors Influencing Response to Oxytocin

**DOI:** 10.3389/fnins.2012.00194

**Published:** 2013-01-10

**Authors:** Kai S. MacDonald

**Affiliations:** ^1^Department of Psychiatry, University of California Medical CenterSan Diego, CA, USA

**Keywords:** oxytocin, sex factors, attachment, oxytocin receptor gene, CD38/ADP-ribosyl cyclase activity

## Abstract

As discussed in the larger review in this special issue (MacDonald and Feifel), intranasal oxytocin (OT) is demonstrating a growing potential as a therapeutic agent in psychiatry. Importantly, research suggests that a variety of individual factors may influence a person’s response to OT. In this mini-review, I provide a review of three: (1) sex and hormonal status; (2) genetic variation in aspects of the OT system (i.e., OT receptors); and (3) attachment history. Each of these factors will be important to monitor as we strive to develop a richer understanding of OT’s role in human development, brain-based disease, and the potential for individualized, OT-targeted treatments.

## Introduction

Aside from a wide range of drug-specific factors (discussed in MacDonald and Feifel in this special edition), several individual factors may influence a person’s response to oxytocin. Three of these factors are reviewed below.

## Sex and Hormonal Status

The central OT system acts as but one component of a complex neurochemical milieu in which gonadal steroids also play a significant part. As extensively discussed in recent full-length reviews, gonadal steroid hormones (i.e., estrogen, progesterone, and testosterone), and the two nonapeptides – OT and arginine vasopressin (AVP) – coevolved, all playing a vital role in mammalian social development through their unique influence on parental bonding, mate choice, and attachment (van Anders et al., 2011; Bos et al., 2012). In toto, there is substantial evidence indicating that at least some of oxytocin’s effects are correlated with an individual’s sex, in part via the influence of gonadal hormones. We can only give this important topic brief review, and direct the reader to more comprehensive treatments (van Anders et al., 2011; Bos et al., 2012; Gabor et al., 2012).

As background, animal studies indicate that sex-specific differences in response to OT are common (Williams et al., 1994; Cho et al., 1999; Bales and Carter, 2003; Bales et al., 2007), and the histological structure for OT neurons is sexually dimorphic, suggesting that sex steroids play a role in early morphogenesis of this system (de Vries, 2008). Estrogen upregulates OT and OT receptor (OTR) production (Patisaul et al., 2003; Windle et al., 2006; Choleris et al., 2008), whereas testosterone promotes both OTR binding in the hypothalamus (Johnson et al., 1991) as well as production of AVP (Delville et al., 1996), which has many opponent actions to OT (Neumann and Landgraf, 2012). In humans, moreover, testosterone seems from one perspective to have opposite behavioral effects to the prosocial impact classically associated with OT: decreasing trust, generosity, empathy (van Honk and Schutter, 2007; Zak et al., 2009; Bos et al., 2010), though more recent conceptualizations of the parochial, “us vs. them” aspect of OT make this picture more complex, and evidence OT’s “darker” side (Shamay-Tsoory et al., 2009; De Dreu et al., 2010, 2011, 2012; Declerck et al., 2010). Though OT is only one small piece of the complex psychobiology of gender, some have posited different OT-biased relational strategies for the sexes, with females more prone to “tend and befriend” (Taylor et al., 2000; but, see Smith et al., 2012b), whereas more warrior-prone, hierarchy-bound males “compete and defeat” (David and Lyons-Ruth, 2005; Smeets et al., 2009; Van Vugt, 2009; Gabor et al., 2012).

More evidence for sex-specific differences in the OT system come from research indicating that men and women show differences in plasma OT levels (Ozsoy et al., 2009; Gordon et al., 2010; Holt-Lunstad et al., 2011; Weisman et al., 2012b), as well as gender-specific behavioral correlations with OT (Gordon et al., 2010; Zhong et al., 2012; but, see Szeto et al., 2011 for critique of plasma OT measurement techniques). Coming from the perspective of genetic variations in nonapeptide receptors, Walum et al. (2012) have found an association between the OTR variant rs7632287 and pair-bonding behaviors in women but not in men, whereas an earlier study found an association of an AVP receptor polymorphism and pair-bonding in *men* but not women (Walum et al., 2008). Furthermore, numerous studies in the growing OTR literature note sex-specific associations between genetic variants in the OTR gene and personality characteristics (Stankova et al., 2012), neural responses to emotionally salient cues (Tost et al., 2010), hypothalamic gray matter volume (Tost et al., 2010), and empathy (Wu et al., 2012), though other studies in this area have failed to find a sex bias (Rodrigues et al., 2009; Saphire-Bernstein et al., 2011; Feldman et al., 2012). A final set of salient investigations found that amygdala-prefrontal cortical connectivity – which can be impacted by OT in normal subjects (Sripada et al., 2012) and anxiety patients (Labuschagne et al., 2011) – may be related in a gender-specific way to the development of anxiety and depressive disorders (Burghy et al., 2012), both putative clinical targets for intranasal oxytocin (IN OT) (Slattery and Neumann, 2010; Neumann and Landgraf, 2012).

Focusing on clinical OT trials using IN OT, gender-dependent effects have been demonstrated in some single-dose studies (Hurlemann et al., 2010), including studies of effects on they amygdala (Domes et al., 2010; Rupp et al., 2012), and interpersonal behavior (Liu et al., 2012) but – consistent with the variability in this literature – many other single-dose studies have not found an effect of sex (see Bartz et al., 2011b for review). A recently investigated individual factor at least partly related to sex (due to different sexual selection strategies between males and females; Ihara and Aoki, 1999) is the relationship status of the person receiving the drug. Specifically, Scheele et al. (2012) found in a group of 86 normal heterosexual males that IN OT preferentially stimulated men in a monogamous relationship – but not single males – to maintain more personal space from women (but not men). Whether these effect would cross over to females and same-sex relationships in interesting and unexplored.

Though the suggestion of gender effects in single-dose studies of normal subjects may be informative, as discussed in the accompanying larger review (MacDonald and Feifel), these results do not speak directly to the clinical question of whether sex differences moderate the effects of chronic OT treatment in clinically ill populations. The first study to intimate such a sex moderation effect was a randomized, double-blind, within-subjects crossover study of OT (40 IU BID for 3 weeks) in patients with generalized anxiety disorder (GAD) (Feifel et al., 2011). This trial demonstrated a trend level dose-by-gender effect such that males treated with OT showed a significant clinical improvement in HAM-A scores with OT, whereas females showed higher HAM-A scores during 3 weeks of treatment. The three extant studies using multiple weeks of OT treatment in patients with schizophrenia demonstrated a male bias in recruitment (62 males treated vs. 13 females), though none showed a sex-by-drug effect (Feifel et al., 2010; Pedersen et al., 2011; Modabbernia et al., 2012). Notable in this context are studies by Rubin et al. (2010, 2011) indicating that female but not male patients with schizophrenia show a correlation between plasma OT concentrations, perception of facial emotion expression, and psychopathology, as well as evidence that women with borderline personality disorder have reduced plasma OT levels, even after controlling for hormonal factors (Bertsch et al., 2012).

In terms of future clinical studies with IN OT, the abovementioned sex-specific variables may have at least two repercussions. First, they highlight the importance of monitoring/measuring hormone levels, menstrual phase, and oral contraceptive status in trials with IN OT, given these parameters may impact OT levels (Salonia et al., 2005, but, see Rubin et al., 2011) and psychiatric symptoms (Rubin et al., 2010). Secondly, given that there are sex differences in the incidence of many of the disease states for which OT is a putative treatment (i.e., autism, postpartum depression), further delineation of the role of sex in the effects of chronic OT treatment will be critical.

## Neuropeptidergic Individuality: Genetic Variations in OTR and CD38

Aside from sex, a second individual factor of import in relation to IN OT treatment involves phenotypically relevant individual genetic variations within different aspects of the OT system (Kumsta and Heinrichs, 2012), what one could call “neuropeptidergic individuality.” This term is annexed from – and a subset of – what Cravchik has called “neurochemical individuality”: genetically determined factors that underlie individual differences in brain function. Exemplars include variations in aspects of the major neurotransmitter systems (i.e., dopamine, serotonin) (Cravchik and Goldman, 2000).

**Figure 1:**
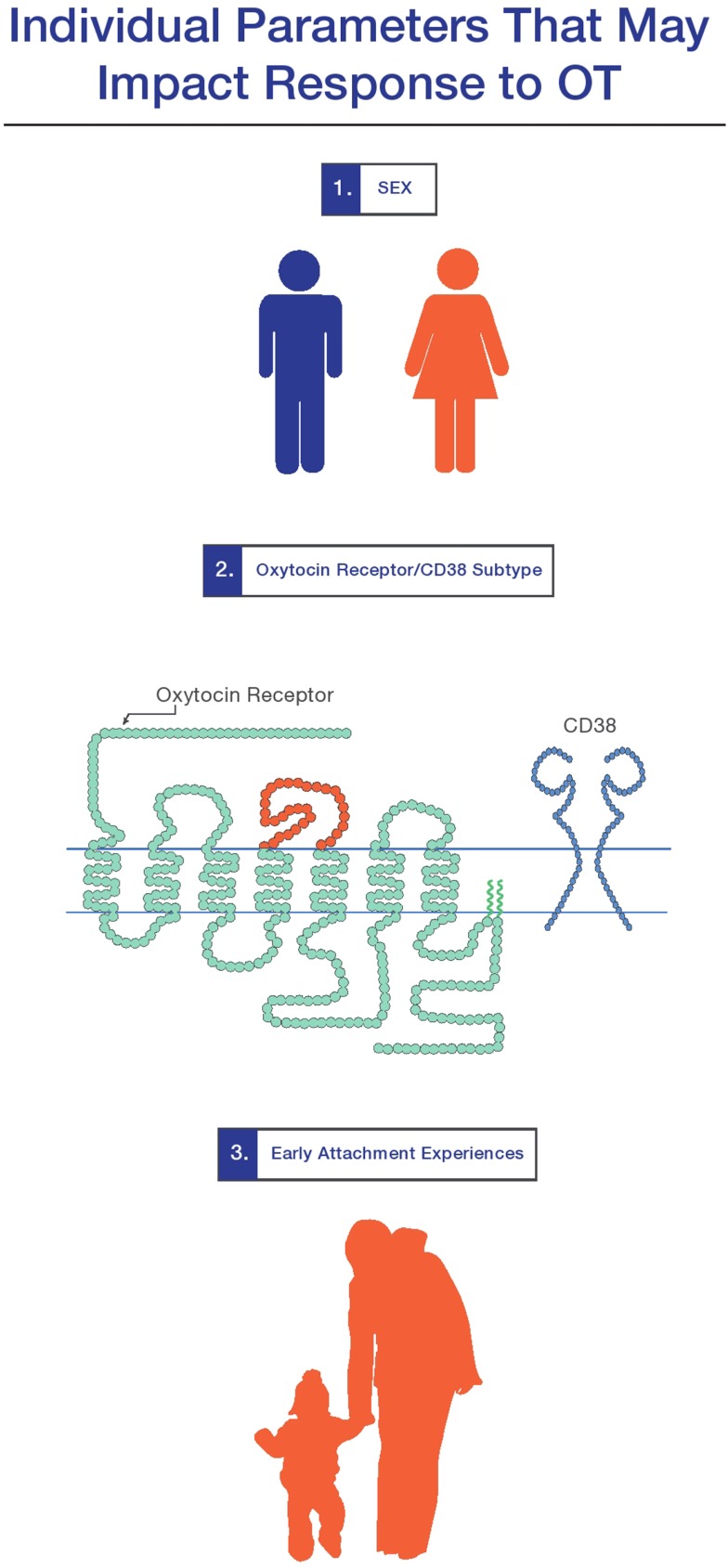
**Three individual factors which mediate response to oxytocin are (1) sex and hormonal status; (2) genetic variations in the oxytocin receptor and CD38 system; and (3) early attachment experiences**. The extent to which these factors play a role in a person’s response to oxytocin-targetted therapeutics for brain-based disease requires further exploration (see MacDonald and Feifel in this special section).

In terms of OTs part in “neuropeptidergic individuality,” a recent, rapidly expanding body of literature indicates that genetic differences in aspects of the functional OT system (the OTR itself and the ectoenzyme CD38, which contributes to OT secretion) (Figure 1) contribute to measurable aspects of an individual’s personality (Kumsta and Heinrichs, 2012). Though the specific cellular and functional consequences of these genetic variations have not been fully explicated, a convergent picture of their phenotypic consequences is emerging, indicating that in neurotypical subjects, genetic differences in the OT system impacts positive personality factors and social behavior (Bakermans-Kranenburg and van Ijzendoorn, 2008; Rodrigues et al., 2009; Montag et al., 2011; Saphire-Bernstein et al., 2011; Walter et al., 2012), differential responses to stress and maltreatment (Kim et al., 2010; Bradley et al., 2011; Chen et al., 2011b; Thompson et al., 2011; Brune, 2012; Norman et al., 2012), brain anatomy (Inoue et al., 2010; Furman et al., 2011), and differences in the function of stress and emotion-related brain areas (Tost et al., 2010; Love et al., 2012). Moreover, genetic variation in the OT system has been implicated in several of the disease states where OT has shown the most therapeutic promise: schizophrenia (Teltsh et al., 2011; Montag et al., 2012) and autism (Ebstein et al., 2012).

Though no published studies have examined the role of genetic variation in the OT system to a psychiatrically ill person’s clinical response to OT, several recent studies in normal subjects indicate that we should be alert for such effects. For example: subjective responses to infant’s faces were moderated by the (rs53576G) allele of the OTR (Marsh et al., 2012); there is an association between several genetic variations in the OTR (rs53576, rs2254298, rs2228485) and performance on the Reading the Mind in the Eyes Test (RMET) (Lucht et al., 2012); and the OXTR (rs2268498) polymorphism modulated neural responses to emotional faces (O’Connell et al., 2012). Moreover, as evidence of the overlap between central dopaminergic and oxytocinergic systems, female OTR (rs4813625) carriers demonstrated greater stress-induced dopamine release, higher attachment and trait anxiety, and lower emotional well-being scores (Love et al., 2012).

A relatively new component of the central OT system – but one which is rapidly galvanizing interest – is the transmembrane enzyme CD38, whose role was discovered by observing the social behavior of CD38 knock-out mice. These socially hapless mice forget the location of their pups as well as previous social encounters, and synthesize, but don’t properly secrete OT. Notably, these behavioral and hormonal deficits are restored with either (a) viral transfection of a functional CD38 gene or (b) exogenous OT (Jin et al., 2007). In humans, variants in the CD38 gene have been tied to OT secretion (Kiss et al., 2011), social processing (Higashida et al., 2012a; Sauer et al., 2012), sensitive parenting (Feldman, 2012), and potentially autism (Higashida et al., 2011, 2012b for review). Similarly to the OTR studies above, a recent imaging genetics study in neurotypical males suggested that variation in the CD38 gene influenced behavioral and neuronal measures of social processing and amygdala response to IN OT (Sauer et al., 2012). A clinical point of interest in this context is that retinoids (vitamin-A related compounds) can be used to increase CD38 expression (Riebold et al., 2011), thus providing an alternative way to stimulate the OT system or potentially augment IN OT treatment (Ebstein et al., 2011).

Though the focus here is only on variation in the OTR and CD38 gene, other potential contributors to neuropeptidergic individuality include: (1) differences in baseline and dynamic levels of OT release in the brain and/or secretion into the plasma, the latter found to correlate with personality and brain structure (Andari et al., 2012); as well as (2) differences in regional OTR and AVPR receptor density, a factor which influences the social behavior of rodents (Hammock and Young, 2006; Ross et al., 2009; Ophir et al., 2012). Though a few studies have examined postmortem OTR density in the human CNS (Loup et al., 1989, 1991), as mentioned in the accompanying review (MacDonald and Feifel), synthesis of a small-molecule radioligand for the OTR (Smith et al., 2012a), would greatly facilitate our understanding of the role of OTR density and location in living humans.

Of critical import in the field of psychiatric genetic association studies are the issues of replicability and effect size (discussed at length in Gershon et al., 2011; Ebstein et al., 2012). For example, in contrast to several of the positive associations noted above, studies have failed to find associations between genetic variations in the OTR and prosocial behavior in the trust or dictator game (Apicella et al., 2010), optimism (Cornelis et al., 2012), and autism (Tansey et al., 2010). Replication studies and larger sample sizes in a variety of populations using different varieties of associations (i.e., different combinations of haplotypes) (Yamasue et al., 2012) are therefore necessary to more fully explore and quantify the strength of the abovementioned associations.

Returning to the clinical implications of neuropeptidergic individuality, it is possible that individual variation in aspects of the OT system may in the future be thought of as clinicians currently conceptualize individual variations in dopamine and serotonin systems. One brings to mind the association of DRD4 variants with approach-related traits (Munafo et al., 2008) and response to dopaminergic medication (Hamarman et al., 2004), or the association of serotonin receptor polymorphisms with susceptibility to adverse clinical outcomes (van Ijzendoorn et al., 2012), as well as response to serotonergic antidepressants (Mrazek et al., 2009). Aside from its import in terms of understanding individual variability in both neurotypical and clinically ill populations, neuropeptidergic individuality may have implications in terms of psychiatric pharmacogenetics: the use of information about individual’s genotype in the selection of psychiatric treatment (Malhotra et al., 2007). Though this approach is currently speculative in terms of OT, it has growing clinically relevance for antidepressants (McMahon et al., 2006) and antipsychotics (Zhang et al., 2010). Looking forward, large clinical trials are needed to investigate the possibility that genetic variations in the abovementioned aspects of the OT system may influence clinical response to OT treatment. That said, the decreasing cost and increasing efficiency of gene sequencing technologies, coupled with larger clinical trials of clinical use of OT (ClinicalTrials.gov), will certainly inform the relevance of this proposed genotype-informed treatment. Moreover, identification of “OT sensitive” phenotypes may optimize patient selection for treatment and trials.

## Early Experience, Epigenetics, and Neuroplasticity

In addition to abovementioned genetically determined factors, a third influence on a person’s response to IN OT concerns the way that that an individual’s unique attachment history has sculpted the function of their OT system (Gordon et al., 2011; Bales and Perkeybile, 2012). More specifically, convergent translational and developmental research in a variety of fields indicates that the central OT system is similar to the HPA axis in being an environmentally influenced plastic brain system whose function is directly and perhaps permanently impacted by early experience (Gunnar and Quevedo, 2008; Brune, 2012; McCrory et al., 2012). Clinically, it is clear that maladaptive early experiences impact the “phenotype” of several psychiatric disorders that may benefit from IN OT, including depression (Saveanu and Nemeroff, 2012) and schizophrenia (Read and Hammersley, 2005; van Os et al., 2010). Recent imaging studies indicate that early adversity impacts brain systems of import to both psychiatric disease and OT treatment (i.e., amygdala and hippocampus; Dannlowski et al., 2012; Teicher et al., 2012).

Research on the environmental plasticity of the OT system began with sentinel animal research indicating intergenerational transmission of behavior in more- and less-attentive rat mothers (Champagne and Meaney, 2001; Champagne et al., 2001; Meaney, 2001). Some of these changes, notably, are mediated via epigenetic modulation of the OT system (Cushing and Kramer, 2005; Stolzenberg et al., 2012). More recently, human experiments support the hypothesis that dynamic changes in components of the OT system (i.e., methylation of the OTR gene; Jack et al., 2012; Unternaehrer et al., 2012) and possibly neurodevelopmental changes in OT sensitive brain structures (see Andari et al., 2012 for discussion) are some of the proximate effectors through which early parental care impacts an individual throughout life (Champagne et al., 2001; Champagne, 2008; Gordon et al., 2011; Bales and Perkeybile, 2012 for reviews). Other convergent evidence comes from attachment-informed behavioral research which indicates parallels and reciprocal influence between parental and infant OT levels and the species-specific behaviors associated with secure attachment and optimal psychosocial development (Feldman, 2012). As mentioned above, these factors appear to be influenced by both genetic variations in the OT system and by IN OT (Naber et al., 2010, 2012; Weisman et al., 2012a).

Focusing specifically on the OT treatment literature, several studies indicate that aversive early attachment experiences and attachment style impact stress systems, CSF, and plasma OT levels (Heim et al., 2009; Strathearn et al., 2009, 2012; Bertsch et al., 2012; Weisman et al., 2012a) as well as later response to IN OT (Huffmeijer et al., 2011, 2012; Simeon et al., 2011; van Ijzendoorn et al., 2011; Bakermans-Kranenburg et al., 2012; Pierrehumbert et al., 2012). For example, neurotypical patients’ generosity in response to IN OT is moderated by parental love-withdrawal (Huffmeijer et al., 2012), and patients with aversive early attachment representations had a negative response to IN OT compared to those with more positive representations (Bartz et al., 2010). Other literature suggests that variation in the OT system may mediate gene-environment interactions between early adversity and outcomes (Kim et al., 2010; Bradley et al., 2011; Chen et al., 2011a; Thompson et al., 2011).

In toto, data reviewed here support the hypothesis that an individual’s early attachment experiences – carried forward in OT-responsive neural networks and the dynamic function of the central OT system – may impact a person’s response to IN OT. To date, in keeping with the general trend noted throughout this and the accompanying larger review (MacDonald and Feifel, this issue) the evidence that early experience impacts OT response in *clinical* populations is sparse. The only published study in this area demonstrated that patients with borderline personality disorder and anxious attachment showed less trust than those with more secure attachment after IN OT (Bartz et al., 2011a). Despite the overall lack of studies of IN OT in patient groups, the findings cited above suggest that clinical trials examining putative therapeutic effects of OT will be wise to include an assessment of attachment style and early trauma as individual factors that may influence response to OT.

## Conclusion

Given the paucity of clinical trials with IN OT, the suggestion that the above factors may be moderators of clinical response to IN OT should be viewed with circumspection. Both larger-scale therapeutic trials with IN OT as well as investigations of the role of aspects of the central OT system in different disease states will be necessary to determine their ultimate clinical and therapeutic relevance.

## Conflict of Interest Statement

The author declares that the research was conducted in the absence of any commercial or financial relationships that could be construed as a potential conflict of interest.
